# Efficiency of Commercial and Test Vaccines Against Lawsonia intracellularis on Clinical and Immunological Parameters in Swine: A Systematic Review

**DOI:** 10.1007/s42770-026-01873-4

**Published:** 2026-04-21

**Authors:** Luiza de Oliveira Possa, Vinícius dos Santos Romagnoli, Renato Lima Senra, Tiago Antônio De Oliveira Mendes

**Affiliations:** Department of Biochemistry and Molecular Biology, Universidade Federal de Viçosa, Viçosa, Brazil

**Keywords:** Lawsonia intracellularis, Swine vaccines, Proliferative enteropathy, Immunological response, Fecal shedding, Weight gain, Mortality rates

## Abstract

*Lawsonia intracellularis* is a gram-negative obligate intracellular bacterium causing Proliferative Enteropathy in pigs, leading to significant economic losses in the swine industry. Vaccination offers an alternative to antibiotics for controlling this pathogen. This systematic review evaluates the efficacy of commercial and experimental vaccines against *Lawsonia intracellularis* in pigs, focusing on clinical outcomes and immunological responses. Following PRISMA guidelines, databases including PubMed, Google Scholar, CABI Digital Library, and Agricola/USDA were searched for studies from the past decade. Selected studies were assessed for bias using SYRCLE’s RoB tool. Data on weight gain, pathogen shedding, mortality, and immune responses were extracted and analyzed. Out of 824 identified articles, 9 studies met the inclusion criteria. Results indicated that vaccinated pigs showed reduced fecal shedding of *L. intracellularis*, lower mortality rates, and enhanced immune responses compared to non-vaccinated controls. Specific vaccines increased IFN-γ production, a crucial cytokine for Th1 responses against intracellular pathogens. Vaccination is an effective strategy to control *Lawsonia intracellularis* infection in swine, reducing disease symptoms and transmission. Continued research is necessary to optimize vaccination protocols and assess long-term economic impacts.

## Introduction

*Lawsonia intracellularis* is a gram-negative, obligate intracellular bacterium that causes Proliferative Enteropathy in pigs. The pathogen is directly associated with economic losses in the global pig production industry due to symptoms such as diarrhea, weight loss, and the costs of prevention and treatment [1]. This bacterium has also been reported in other species, such as horses and primates [2, 3]. Porcine Proliferative Enteropathy can be divided into three classes: acute, chronic, and subclinical, each with distinct clinical signs and characteristics [4]. The acute form is characterized by hemorrhagic diarrhea and anemia, which can lead to death, and is most common in animals aged 4–12 months. Chronic form is associated with poor growth accompanied by diarrhea without visible mucus or hemorrhage, while the subclinical form shows fewer clinical signs, such as normal feces and slight difficulty in gaining weight, as well as smaller and less severe intestinal lesions [4–6].

Infection by *Lawsonia intracellularis* in pigs requires appropriate prevention and treatment to control the pathogen. The classic method of control is based on therapieutic treatment with oral administration of antibiotics to combat the infection and reduce symptoms [7]. Tilvalosin, tylosin, tiamulin, and oxytetracycline are generally the drugs of choice for treatment of Porcine Proliferative Enteropathy [8–11]. However, despite their effectiveness, the excessive use of antibiotics results in several issues, such as high associated costs and the risk of emergence of resistant pathogen strains in the environment [12, 13].

In this context, vaccination is a control method that demonstrates efficacy and safety against Porcine Proliferative Enteropathy. Vaccine can be efficient for both preventing *L. intracellularis* infection and in reducing transmission and the economic losses caused by the disease [10]. In this study, we conducted a systematic review to evaluate the efficacy of commercial and published candidates vaccines tested in pigs over the past 10 years. The vaccine studies are were dissected to recover information about weight gain, pathogen shedding in feces, mortality rate, and the immune response of animals subjected to immunization against *L. intracellularis*. This systematic review aims to compile data and facilitate understanding of the current situation related to the prevention of Porcine Proliferative Enteropathy in other to provide valuable information to support new studies in the field.

## Materials and methods

The protocol of this systematic review and reporting of the results followed the recommendations described by the Preferred Reporting Items for Systematic Reviews and Meta-Analyses (PRISMA, 2020) [14].

### Search and selection criteria

The searches for articles were performed in four databases, U.S. National Library of Medicine and the National Institutes of Health (PubMed), CABI Digital Library, Agricola/USDA and Google Scholar, and articles published in English in the last 10 years were used. For the four databases, the terms used for the searches were: (lawsonia* OR lawsonia intracellularis OR l. intracellularis OR swine proliferative enteropathy OR ileitis) AND (vaccine OR immuni* OR vaccin* OR interve* OR treatment OR efficacy OR effect OR control* OR protect OR shed OR swine diseases) AND (swine OR pig OR pigs OR piglet OR piglets OR porcine OR pork OR gilt OR gilts OR sow OR sows OR hog OR hogs). The asterisk was used to broaden the search for related words and terms.

### Screening and eligibility of studies

The search terms followed the PICOS strategy (Table 1). The searches were carried out by two researchers independently, and the divergences were discussed and decided by consensus. Only original articles investigating the protective efficacy of existing vaccines and vaccine candidates and their effects on the immune response and clinical parameters of pigs against *Lawsonia intracellularis* were considered. In addition, the references of all selected articles were analyzed to identify possible relevant studies for inclusion in the review.

**Tabela 1:** PICOS criteria for inclusion and exclusion of studies for a systematic review

Acronym	Parameter	Definition
P	Population	Porcine
I	Intervention	Vaccination (commercial or candidate)
C	Comparison	Negative control porcine (unvaccinated or simulated vaccinated
O	Outcomes	Lower mortality, fecal pathogen excretion, and diarrhea; Increased weight gain and improved immune markers.
S	Study design	Experimentally vaccinated pigs were included. Other animal or human species, letters to the editor, or studies that did not evaluate vaccines or vaccine candidates were excluded.

*PICOS*: Population, Intervention, Comparison, Outcomes, Study design

After the searches, duplicate articles were excluded and the initial screening of articles was performed by title and abstract. In addition, articles that did not present the main theme, reviews, papers on other animal or human species, letters to the editor, or studies that did not evaluate vaccines or vaccine candidates were also excluded. The selected studies were read in full and evaluated for eligibility criteria.

### Data extraction and synthesis

**Fig. 1:**
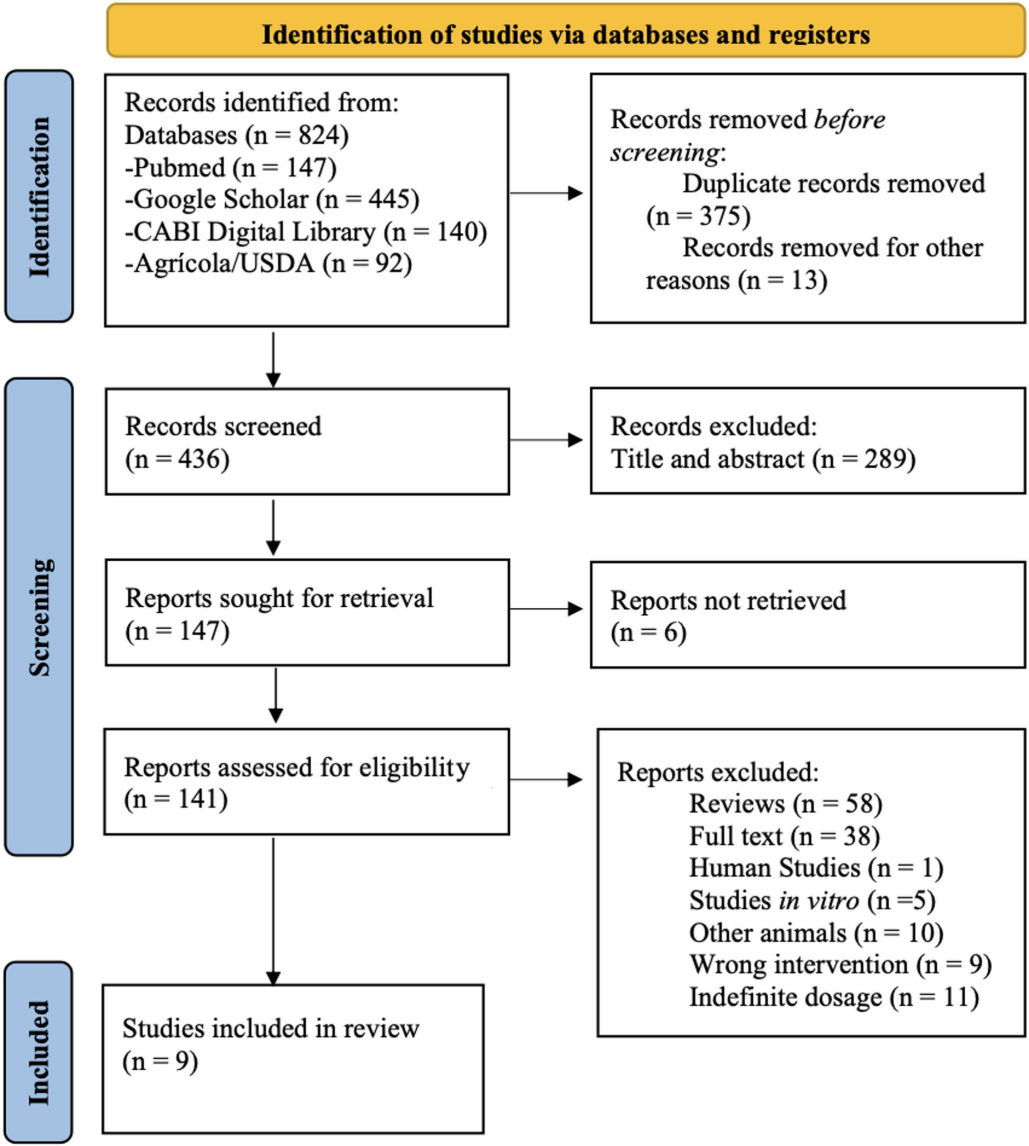
Flow diagram of the studies selection process.

The information of interest of the studies was extracted and consisted of: (1) general characteristics, including title, authors, year of publication, country; (2) characteristics of the animal model such as lineage, number of animals evaluated (vaccinated and controls), age, sex, mean initial weight; (3) characteristics of the intervention such as type of vaccine and treatment, dose, duration of intervention, frequency, route of administration, challenge, and dose; (4) Findings including fecal elimination, mortality rate, weight gain or loss, and immune response (Fig. 1). Histopathological results of the studies were not evaluated due to the risk of observer bias.

### Risk of bias assessment

**Fig. 2:**
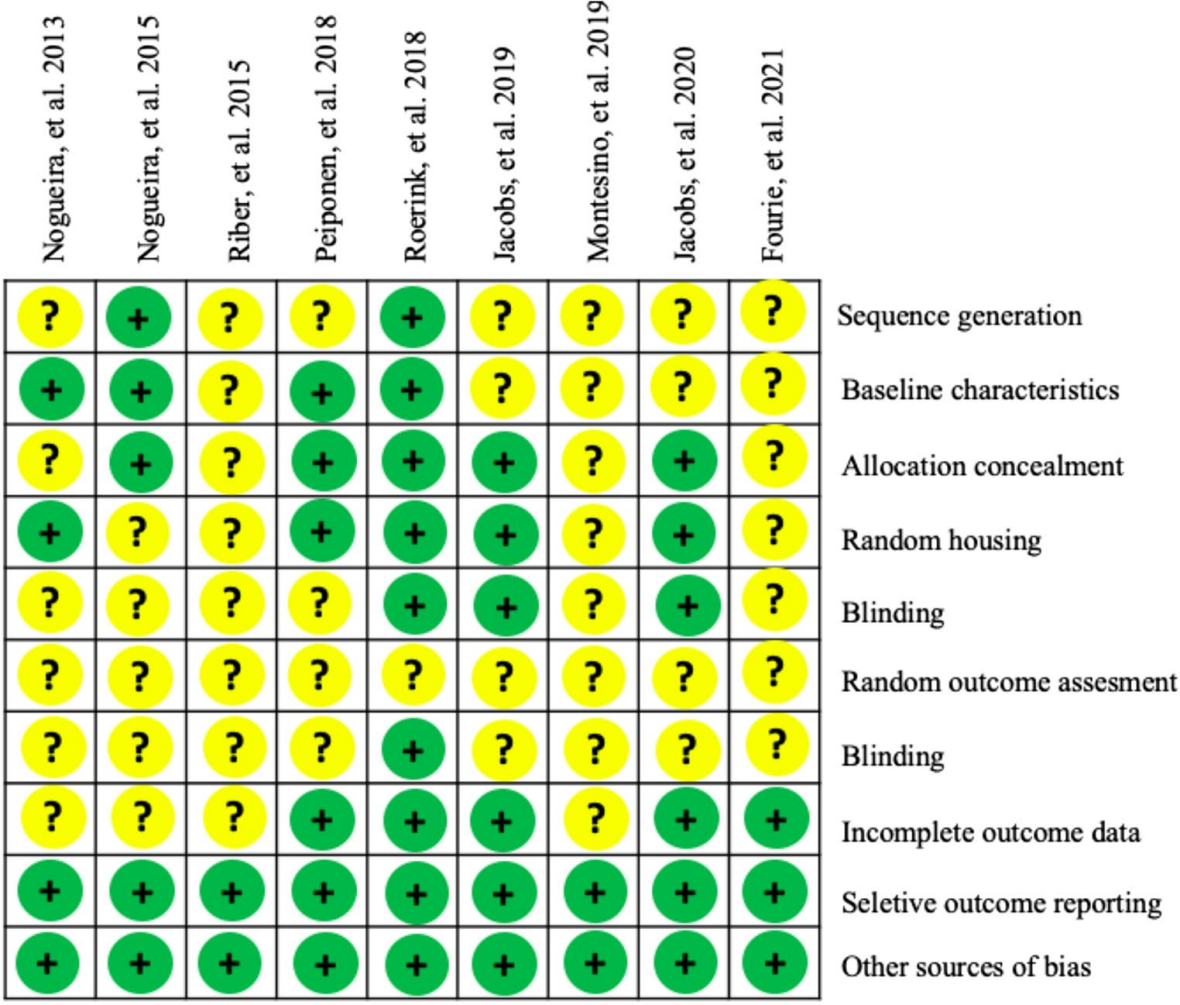
SYRCLE's Risk of Bias: authors' assessment for the included studies regarding each risk of bias item

The articles selected for this review were analyzed for risk of bias according to the guidelines of the SYRCLE’s RoB tool (Fig. 2). Two authors (V. S. R. and R. L. S.) independently conducted the analyses, with decisions made by consensus. Discrepancies were reviewed by a third reviewer (L. O. P.). The results of the risk of bias analysis were classified as low risk, high risk, and unclear.

## Results

### Selected studies

The flow diagram for the selection of articles was completed according to the PRISMA guideline, including the steps of search, identification, screening and selection of studies (Fig. 1). A total of 824 articles were retrieved from the four databases: Pubmed (*n* = 147); Google Scholar (*n* = 445); CABI Digital Library (*n* = 140) and Agricola/USDA (*n* = 92). Of these articles, eight hundred and twelve (*n* = 812) articles were excluded, and the main criteria were duplicate articles (*n* = 375), title and abstract outside the scope of this review (*n* = 289) and review text type (*n* = 58). The other criteria used to exclude articles are described in the flowchart. At the end of the stages, nine (*n* = 9) [15–23] articles met the inclusion criteria for this review.

The selected articles had their experiments conducted in 7 different countries. The most utilized breed was Landrace/Large White (44.4%, *n* = 4), followed by Yorkshire/Duroc; however, three studies did not mention the breed of the animals. The age of the animals at the beginning of the study ranged from 3 to 6 weeks, with sample sizes varying from 8 per group to 1642 animals. The average initial weight of the animals was reported in 33.3% of the studies (*n* = 3) (Table 2).

**Tabela 2:** Characteristics of selected studies on the protective efficacy of vaccines and their effects on the immunological response and clinical parameters of pigs against *Lawsonia intracellularis*

References	Country	Breed	Sample size	Age (weeks)	Weight
Nogueira, et al. 2013 [15]	Australia	Landrace/Large White	40	5	5,5–6,5 kg
Nogueira, et al. 2015 [16]	Australia	Landrace/Large White	20	4	-
Riber, et al. 2015 [17]	Denmark	Danish Landrace-Yorkshire/Duroc	25	5–6	8–12 kg
Peiponen, et al. 2018 [18]	Finland	-	1642	3	6–7 kg
Roerink, et al. 2018 [19]	United States	Mixed race	85	3	-
Jacobs, et al. 2019 [20]	Netherlands	-	75 (per assay)	4–5	-
Montesino, et al. 2019 [21]	Chile	Duroc/Yorkshire	24	4	-
Jacobs, et al. 2020 [22]	Netherlands	-	75a/50b	3	-
Fourie, et al. 2021 [23]	Canada	Landrace/Large White	8a/32b	5	-

^a^- Study 1, ^b^- Study 2, *Kg* kilogram

Regarding vaccination, seven studies used commercially available vaccines, with four of them employing live attenuated vaccines. Additionally, seven studies administered the same volume of vaccine formulation (2mL), while the smallest dose was 0.2mL. The primary route of administration was intramuscular, followed by oral vaccination, either alone or in combination with other routes. Challenge was predominantly conducted through infected intestinal mucosa with *L. intracellularis* in most studies. The majority of studies used 20 to 40mL of inoculum, with two studies not specifying the volume administered. To assess the effect, studies included non-vaccinated control groups (*n* = 8), non-challenged controls (*n* = 2), or those vaccinated with another commercial vaccine (*n* = 2), either individually or combined within the same study. The shortest evaluation and follow-up period in the studies was 17 days, while the longest was 27 weeks. Euthanasia in most studies (*n* = 4) occurred 21 days after challenge (Table 3).

**Tabela 3:** Methodologies utilized in studies on the protective efficacy of vaccines and their effects on the immunological response and clinical parameters of pigs against *Lawsonia intracellularis*

References	Treatment		Dose	Route	Challenge	Grupos	Control	Time	Euthanasia
	Commercial	Candidate							
Nogueira, et al. [15]	Ileíte Enterisol^®^		2mL	Oral and IM	4 weeks post vaccination −25mL of infected intestinal mucosa (109)	5	Unvaccinated	6 weeks	21 days post challenge
Nogueira, et al. [15]	Ileíte Enterisol^®^		2mL	Oral, IM and IP	-	4	Unvaccinated	17 days	17 days post vaccine
Riber, et al. [17]	Ileíte Enterisol^®^		2mL	Oral	12 a 13 weeks for age of -Infected intestinal mucosa (1010)	3	Não infected	84 days	5 weeks post challenge
Peiponen, et al. [18]	Enterisol^®^ Ileite e Ingelvac CircoFLEX^®^		2mL (Oral) 1mL (IM)	Oral and IM	-	2	Unvaccinated and vaccinated for Circovírus	86 days	Day 86
Roerink, et al. [19]	Porcilis^®^Ileite		2mL	IM	5 weeks post vaccination −25mL of infected intestinal mucosa (109)	3	Unvaccinated	51 days	21 and 51 days post challenge
Jacobs, et al. [20]	Porcilis^®^ Lawsonia		2mL	Oral and IM	4a, 17b e 3c weeks post vaccination −20mL of infected intestinal mucosa	3a^, b, c^	Unvaccinated	11 days	21 days post challenge
Montesino, et al. [21]		Chimeric antigen pLawVac	1mL	IM	7 weeks post vaccination − 40 mL of infected intestinal mucosa	3	Unvaccinated and vaccinated with suine IFN-α	11 weeks	30 days post challenge
Jacobs, et al. [22]	Porcilis^®^ Lawsonia ID e Porcilis^®^ PCV ID		0,2mL	Identificação	4 and 21 weeks post vaccination - Infected intestinal mucosa	5 (3a/2b)	Unvaccinated	14 weeks/ 27 weeks	21 days post challenge
Fourie, et al. [23]		rTgD, rFliCa NEVOEIRO, CMb	2mL	IM	Dia 27 −40mL of infected intestinal mucosa (2,0 × 108)	5 (2a/3b)	Unvaccinated Challenged/unchallenged	6 weeks	Day 46

^a^ - Study 1, ^b^ - Study 2, ^c^ - Study 3, *ID* intradermal, *IM* intramuscular, *IP* intraperitoneal, *FOG* rFliC, rOppA, rGroEL, *CM* rClpP, rMetK, *mL* milliliter

### Weight control

Seven selected publications assessed weight control as an outcome of interest. Among these, 4 studies showed no statistically significant differences in weight gain or loss between control and test groups, with variations of less tham 100 g per day [15, 17, 21, 23]. Peiponen et al. (2018) reported higher weight gain of 3.57 kg at slaughter in vaccinated animals compared to the non-vaccinated group. During the nursery phase, average weight gain was 14.8 g per day, and during the finishing phase, it reached 30.9 g per day [18]. Jacobs et al. (2020) reported, in their first study, average daily gains between 13 and 20 days post-challenge of 956 g/day in animals vaccinated with the inactivated intradermal vaccine, compared with 812 g/day in animals vaccinated with the oral live vaccine, and 674 g/day in the non-vaccinated group. In their second study, the same authors observed an average daily gain of 1001 g/day in animals vaccinated with the intradermal formulation combined with the PCV ID vaccine, whereas non-vaccinated animals showed an average weight loss of 139 g/day. Similar findings were reported by Jacobs et al. (2019). In their first study, the authors found an average daily gain of 649 g/day in animals vaccinated with the inactivated intramuscular vaccine, while animals vaccinated with the live vaccine gained 537 g/day, and the non-vaccinated group showed a marked weight loss of 655 g/day after challenge. In the second study, the average daily gain between 13 and 20 days post-challenge was 1012 g/day in vaccinated animals, compared with 537 g/day in the non-vaccinated control group. In the third study, the average daily weight gain in animals vaccinated with the formulation combined with PCV M Hyo was 649 g/day, while non-vaccinated animals again showed a post-challenge weight loss of 655 g/day [20, 22].

### Fecal elimination

Of the nine selected studies, six assessed the shedding and load of *Lawsonia intracellularis* in the feces of the tested pigs. All studies that investigated this outcome reported higher pathogen excretion in the non-vaccinated groups compared with the vaccinated groups, and four of them provided quantitative data that allow for comparative analysis. Riber et al. (2007) observed that non-vaccinated pigs showed fecal loads ranging from 10⁷ to 10⁸ copies of *L. intracellularis* DNA per gram of feces, whereas immunized animals maintained levels between 10⁵ and 10⁶ DNA copies per gram throughout the post-challenge period, representing a reduction of approximately 1.2 to 1.7 log₁₀ in bacterial shedding [17]. Similarly, Fourie et al. (2021) demonstrated that the multivalent FOG vaccine reduced fecal shedding by about 68% on day 11 post-challenge and 71% on day 18, compared with the control group, while the CM formulation showed smaller reductions (6–11%) [23].

In the studies by Jacobs et al. (2019, 2020) that assessed *Lawsonia intracellularis* shedding using qPCR, consistently higher pathogen excretion was observed in the non-vaccinated groups. In one of the experiments, vaccinated groups exhibited fecal loads between 0.23 and 0.34 log₁₀ pg DNA/µL at 21 days post-challenge, whereas the non-vaccinated group reached 0.60 log₁₀ pg DNA/µL [20]. In another study conducted in the same year, vaccinated animals showed even lower loads, ranging from 0.11 to 0.27 log₁₀ pg/µL, while the non-vaccinated controls presented 0.46 log₁₀ pg/µL. In a third experimental set, the combined vaccine formulation resulted in 1.37 log₁₀ pg/µL, whereas higher values were recorded both in animals vaccinated with the live vaccine (2.13 log₁₀ pg/µL) and in the non-vaccinated pigs, which exhibited the highest shedding at 2.47 log₁₀ pg/µL [22].

These findings demonstrate that, across all evaluated scenarios, fecal loads were substantially lower in the vaccinated groups, confirming the reduction in *L. intracellularis* shedding promoted by vaccination (Table 4).

**Tabela 4:** Main results of selected studies on the protective efficacy of existing vaccines and candidate vaccines and their effects on the immunological response and clinical parameters of pigs against *Lawsonia intracellularis*

References	Weight control	Fecal elimination	Mortality	Immune response	Others
Nogueira, et al. 2013 [15]	No difference in weight gain between groups.	Significantly lower fecal shedding and bacterial load in the oral vaccinated group.	-	↑ specific IgG and anti-IgG in the oral and IM vaccinated groups. ↑ IgM, IFN-γ, IL-6, IL-10, TNF-α e TGF-β1 in the oral vaccinated group.	-
Nogueira, et al. 2015 [16]	-	-	-	↑ specific IgG in the oral vaccinated group. ↑ mucosal IgG and IgA for oral and IP vaccinated groups and mucosal IgG for IM vaccinated group. ↑ TNF-α e TGF-β1 for oral and IP vaccinated groups.	-
Riber, et al. 2015 [17]	No difference in weight gain between groups.	Significantly lower fecal shedding and bacterial load in vaccinated groups compared to unvaccinated groups. Shedding of 105-108 L.intracellularis/g stool in infected group.	-	↑ specific IFN-γ in vaccinated and unvaccinated animals post challenge. ↑ antigen-specific T cells before challenge ↑ TCD4^+^CD8^+^ cells in the previously infected group, compared to the vaccinated and unvaccinated groups.	Detection of *L. intracellularis* in the ileum of 2 vaccinated animals and ileum and lymph nodes of 7 unvaccinated pigs.
Peiponen, et al. 2018 [18]	Greater weight gain in vaccinated animals compared to the unvaccinated group.	-	No difference in mortality rate between groups.	-	-
Roerink, et al. 2018 [19]	-	↓ Bacterial shedding in 15 Diarrhea in 40% of animals in the unvaccinated group.	-	↑ anti-Lawsonia antibodies in vaccinated pigs before challenge, compared to control.	↓ appetite in the control group. 28% of the animals in the control group had severe ileitis.
Jacobs, et al. 2019 [20]	Suboptimal weight gain in animals in the non-vaccinated group. Vaccinated animals showed significant reduction in weight loss.	Lower fecal shedding and bacterial load in the vaccinated group.	↓ mortality associated with *L. intracellualris*	antibodies post vaccination in vaccinated groups	↑lesions in the ileum (redness and mucosal thickening) ↓ clinical signs in vaccinated animals
Montesino, et al. 2019 [21]	No difference in weight gain between groups	-	No animals died during the experimental period.	↑ humoral immune response pIFN-α combined with *L. intracellularis* antigens. ↑ oas2 mRNA in the group with the formulation containing pIFN-α	Mucosal thickening
Jacobs, et al. 2020 [22]	- Suboptimal weight gain in animals in the non-vaccinated group - Vaccinated animals with Porcilis® Lawsonia ID showed a significant increase in weight gain and reduction of negative clinical signs.	Significantly lower fecal shedding and bacterial load in vaccinated groups compared to unvaccinated groups	4a and 3b animals died or were slaughtered for causes unrelated to the experiment	↑ antibodies responses 3 weeks post challenge in vaccinated group with Porcilis® Lawsonia ID compared to the other groups ^a^ ↑ antibodies in vaccinated group with Porcilis® Lawsonia ID compared to the other groups^b^	↑lesions in the ileum (redness and mucosal thickening)^a^ ↓ diarrhea, weight loss and shedding of L. intracellularis in stool.^b^
Fourie, et al. 2021 [23]	No difference in weight gain between groups	Lower fecal shedding and bacterial load in the FOG-vaccinated group	2 animals were slaughtered for causes unrelated to the experiment	rFliC^a,b^, rGroEL^b^ and rClpP^b^ vaccines induced antibody and mucosal immune responses in vaccinated animals compared with the control group.	

↑ increase, ↓ reduction, *IgA* immunoglobulin A, *IgG* immunoglobulin G, *IgM* immunoglobulin M, *IFN-γ* interferon gamma protein, *IL-6* interleukin 6, *IL-10* interleukin 10, *TNF-α* tumor necrosis factor-alfa, *TGF-β1* transforming growth factor beta 1, *mRNA* messenger ribonucleic acid, %: percent

### Mortality

Four of the selected studies reported mortality outcomes in their trials. In the study by Peiponen et al. (2018), nursery-phase mortality was 6.5% (64/986) among vaccinated pigs and 5.6% (55/983) in the control group, while finishing-phase mortality was identical between groups, reaching 1.2% in both vaccinated and non-vaccinated animals [18]. One study reported a clear difference in mortality between the evaluated groups. In the vaccinated group, no animals died as a result of *Lawsonia intracellularis*, resulting in 0% mortality associated with ileitis. In contrast, the non-vaccinated group had 11 deaths attributed to the infection, indicating a strong clinical impact of the disease in the absence of immunization [20]. In the study reported by Jacobs et al. (2020), mortality rates also demonstrated a clear advantage for vaccinated animals. Mortality specifically associated with *Lawsonia intracellularis* was 0.45% in immunized animals, whereas it reached 1.65% in non-vaccinated animals [22]. In one study, no mortality associated with the challenge or with the vaccines was observed [23].

### Immune response

Eight of the nine selected studies evaluated the immune response of the tested animals. All of these studies reported a higher humoral immune response in vaccinated animals, with increased production of IgG, IgA, and IgM specific to *Lawsonia intracellularis.* Two studies assessed the IFN-γ response and demonstrated an increase in this cytokine when the animals were vaccinated [15, 17]. Two studies evaluated the TNF-α and TGF-β1 responses, also reporting increased production of these cytokines in the vaccinated groups [15, 16]. One study assessed IL-6 and IL-10, reporting increased levels in the vaccinated groups [15]. Riber et al. (2015) reported an increase in the population of CD4 + and CD8 + T cells in the group previously infected with the pathogen, compared to the control and vaccinated groups [17].

## Discussion

The present study aimed to investigate the effects of vaccinating pigs against *Lawsonia intracellularis* on outcomes related to production indicators, pathogen transmission, and immune response to the vaccine formulation. Given that this is an endemic disease worldwide with a high infectious capacity, it causes financial losses for producers, justifying the search for increasingly efficient vaccine candidates. One of the main causes of loss is the ability of *Lawsonia intracellularis* infection to interfere with animal growth performance, especially during the growing and finishing phases [4, 24]. In this study, four selected publications did not demonstrate significant differences in weight gain between vaccinated and non-vaccinated animals during the study period [15, 17, 21, 23]. which can be explained by the limited duration of the experiments, not accurately reflecting the growing and finishing phases. The inefficient weight gain in the non-vaccinated groups reported by the other three selected studies [18, 20, 22]; can be explained by intestinal lesions, specifically in the ileum and jejunum, which reduce nutrient absorption and consequently, animal performance [25, 26].

Due to the shedding of the intestinal epithelium infected with *Lawsonia intracellularis*, it is expelled along with fecal content, being one of the main ways of pathogen transmission, as well as an important method for detecting infected animals [27, 28]. Thus, controlling the shedding of *Lawsonia intracellularis* in feces is a way to contain pathogen transmission among animals. Among the articles selected for this study that evaluated *Lawsonia intracellularis* shedding in feces, all reported a reduction in the bacterial load shed by vaccinated animals compared to non-vaccinated groups [15, 17, 19, 20, 22, 23]. These results demonstrate that vaccination is an effective way to prevent the spread of the pathogen in the environment and reduce the exposure of healthy animals to the risk of contamination.

Vaccination against Porcine Proliferative Enteropathy presents a mixed immune response pattern, with systemic humoral response, mucosal humoral response, and cellular immune response present [29]. The systemic humoral response is mediated by the production of IgG and IgM antibodies, while the mucosal humoral response is mediated by the production of IgA specific to *Lawsonia intracellularis.* This response to vaccination described in the selected articles is important to prevent infection before the pathogen lodges in the enterocytes and is shed in the feces. Another important response to vaccination described in the selected studies is the increased expression of IFN-γ and TNF-α, corroborating with other authors. Hidalgo-Gajardo et al. (2023) analyzed the cellular immune response of pigs immunized with different formulations of a recombinant protein with or without *Lawsonia intracellularis* antigens [30]. The expression of TNF-α was significantly higher in the group immunized with protein plus pathogen antigen on day 5 compared to other groups. The expression of IFN-γ was also higher in the recombinant protein plus antigens group and the recombinant protein group on days 5 and 15, respectively, after vaccination. These results are also in agreement with Salazar et al. (2023) [31]. The authors reported a significant increase in IFN-γ production in groups immunized with the developed vaccine candidate, compared to non-immunized controls and those immunized with a commercial vaccine, after 6 and 14 weeks. These cytokines are essential for an effective Th1 cellular response to combat intracellular pathogens such as *Lawsonia intracellularis.* Although the humoral response, with the production of specific antibodies, is important, a greater Th1 polarization induced by vaccination through increased IFN-γ tends to be more effective due to the pathogen’s survival mechanisms.

The use of recombinant antigens in the prevention of porcine proliferative enteropathy is still in its early stages and underutilized compared to live attenuated vaccines. Currently, there are no approved recombinant vaccines available for field use. It is crucial to conduct further research to develop and evaluate new recombinant protein antigens, as this technology holds significant potential for vaccine efficacy due to the pathogen’s infection mechanisms and life cycle. Additionally, recombinant antigen production is cost-effective, highly scalable, and safe.

## Conclusion

Thus, vaccination stands at the fore as an alternative approach to managing *Lawsonia intracellularis* infection in pigs rather than using antibiotics. The benefits from vaccination include the enhancement of disease resistance and reduction of clinical symptoms which are augmented by immunological effect and other health parameters in swine herds. The topic continues to need more research coupled with field evaluations that should be conducted to better define optimal vaccination strategies and understand downstream economic and long-term positive impacts of this approach on the swine health system. The goal of this review is not only to inform future studies but also assist researchers in understanding key issues related to the subject matter by providing a brief description and critical analysis of major published works within this area.
